# Retraction: Presence of Contagious Bacterial Flora in Formalin-Fixed Cadavers: A Potential Health Hazard to Medical Professionals

**DOI:** 10.7759/cureus.r198

**Published:** 2025-11-04

**Authors:** Ppavani Gundreddy, Sagar S Gaurkar

**Affiliations:** 1 Anatomy, Jawaharlal Nehru Medical College, Datta Meghe Institute of Medical Sciences, Wardha, IND; 2 Otolaryngology - Head and Neck Surgery and Surgical Oncology, Jawaharlal Nehru Medical College, Datta Meghe Institue of Medical Sciences, Wardha, IND

This article has been retracted by the Editors-in-Chief due to plagiarism. Data and findings from the following article have been falsely presented as the original work of the authors: Tabaac B, Goldberg G, Alvarez L, Amin M, Shupe-Ricksecker K: Bacteria detected on surfaces of formalin fixed anatomy cadavers. IJAE: Italian Journal of Anatomy and Embryology: 118 (1), 2013, 1-5. https://oajournals.fupress.net/index.php/ijae/article/view/1138/1136

